# Beyond ecosystem modeling: A roadmap to community cyberinfrastructure for ecological data‐model integration

**DOI:** 10.1111/gcb.15409

**Published:** 2020-11-06

**Authors:** Istem Fer, Anthony K. Gardella, Alexey N. Shiklomanov, Eleanor E. Campbell, Elizabeth M. Cowdery, Martin G. De Kauwe, Ankur Desai, Matthew J. Duveneck, Joshua B. Fisher, Katherine D. Haynes, Forrest M. Hoffman, Miriam R. Johnston, Rob Kooper, David S. LeBauer, Joshua Mantooth, William J. Parton, Benjamin Poulter, Tristan Quaife, Ann Raiho, Kevin Schaefer, Shawn P. Serbin, James Simkins, Kevin R. Wilcox, Toni Viskari, Michael C. Dietze

**Affiliations:** ^1^ Finnish Meteorological Institute Helsinki Finland; ^2^ Department of Earth and Environment Boston University Boston MA USA; ^3^ School for Environment and Sustainability University of Michigan Ann Arbor MI USA; ^4^ Biospheric Sciences Laboratory (618) NASA Goddard Space Flight Center Greenbelt MD USA; ^5^ Earth Systems Research Center University of New Hampshire Durham NH USA; ^6^ ARC Centre of Excellence for Climate Extremes Sydney NSW Australia; ^7^ Climate Change Research Centre University of New South Wales Sydney NSW Australia; ^8^ Evolution & Ecology Research Centre University of New South Wales Sydney NSW Australia; ^9^ Department of Atmospheric and Oceanic Sciences University of Wisconsin‐Madison Madison WI USA; ^10^ Harvard Forest Harvard University Petersham MA USA; ^11^ Jet Propulsion Laboratory California Institute of Technology Pasadena CA USA; ^12^ Department of Atmospheric Science Colorado State University Fort Collins CO USA; ^13^ Computational Earth Sciences Group and Climate Change Science Institute Oak Ridge National Laboratory Oak Ridge TN USA; ^14^ Department of Civil and Environmental Engineering University of Tennessee Knoxville TN USA; ^15^ Department of Organismic and Evolutionary Biology Harvard University Cambridge MA USA; ^16^ NCSA (National Center for Supercomputing Applications) University of Illinois at Urbana Champaign Urbana IL USA; ^17^ College of Agriculture and Life Sciences University of Arizona Tucson AZ USA; ^18^ The Fulton School at St. Albans St. Albans MO USA; ^19^ Natural Resource Ecology Laboratory Colorado State University Fort Collins CO USA; ^20^ UK National Centre for Earth Observation and Department of Meteorology University of Reading Reading UK; ^21^ Fish, Wildlife, and Conservation Biology Department Colorado State University Fort Collins CO USA; ^22^ National Snow and Ice Data Center Cooperative Institute for Research in Environmental Sciences University of Colorado Boulder CO USA; ^23^ Brookhaven National Laboratory Environmental and Climate Sciences Department Upton NY USA; ^24^ University of Delaware Newark DE USA; ^25^ Ecosystem Science and Management University of Wyoming Laramie WY USA

**Keywords:** accessibility, benchmarking, community cyberinfrastructure, data, data assimilation, ecosystem models, interoperability, reproducibility

## Abstract

In an era of rapid global change, our ability to understand and predict Earth's natural systems is lagging behind our ability to monitor and measure changes in the biosphere. Bottlenecks to informing models with observations have reduced our capacity to fully exploit the growing volume and variety of available data. Here, we take a critical look at the information infrastructure that connects ecosystem modeling and measurement efforts, and propose a roadmap to community cyberinfrastructure development that can reduce the divisions between empirical research and modeling and accelerate the pace of discovery. A new era of data‐model integration requires investment in accessible, scalable, and transparent tools that integrate the expertise of the whole community, including both modelers and empiricists. This roadmap focuses on five key opportunities for community tools: the underlying foundations of community cyberinfrastructure; data ingest; calibration of models to data; model‐data benchmarking; and data assimilation and ecological forecasting. This community‐driven approach is a key to meeting the pressing needs of science and society in the 21st century.

## Graphical abstract

Model‐data integration demands of the ecological community, managers, policymakers and the society are becoming increasingly diverse and complex. A new strategy is needed to approach challenges in reducing the informatics bottlenecks to process ecological information, involving the whole research community to play an active role in confronting models with data and advancing our ecological understanding. We argue that a major step towards achieving ecological model‐data integration in a way that is transparent, easily communicable, and scales up to the size and diversity of the ecological community lies in the development and support of community‐wide cyberinfrastructure.

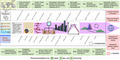

## 1 INTRODUCTION

Kindled by rapid environmental change, the scientific community is deeply invested in understanding and predicting nature's dynamics (Dietze et al., 2018; Hanson & Walker, 2020; Rineau et al., 2019). Thankfully, recent decades have seen an explosion of environmental data globally that is being delivered to us faster than ever before (Farley et al., 2018; LaDeau et al., 2017; Reichstein et al., 2019; Schimel et al., 2019). Process‐based ecosystem models play a critical role in translating data into mechanistic understanding, as they provide us with the ability to synthesize and reformulate knowledge across organizational, spatial, and temporal scales, and to generate testable predictions from alternative hypotheses (Fisher et al., 2014; Hanson & Walker, 2020; Medlyn et al., 2015). Despite having more data than ever before, we have not seen comparable progress in our capacity to forecast natural systems with process‐based models (Bonan & Doney, 2018; Dietze et al., 2018; Lovenduski & Bonan, 2017). For example, model projections out to the year 2100 do not agree on whether terrestrial ecosystems will be a carbon sink or source in response to climate change, and these discrepancies have not changed despite years of apparent model improvement (Arora et al., 2020; Friedlingstein et al., 2006, 2014). Perhaps this is not unexpected: adding model complexity without being informed by data does not equate to improved predictions, new processes (e.g., nutrients) may increase realism but may undo previous calibrated performance unless calibration is renewed easily. Overall, it is not a simple task to evaluate multiple model ensembles, making conclusions about forecast capacity complicated (Herger et al., 2019; Lovenduski & Bonan, 2017). A new strategy is needed to approach challenges in advancing our ecological understanding, reducing uncertainties, and integrating the disparate science communities of global change biology (Bonan & Doney, 2018; Dietze et al., 2018). The goal of this paper is to better characterize the bottlenecks that have obstructed the rates at which new information has been integrated into ecosystem models, and to lay out a roadmap to overcome these bottlenecks. While many of the examples here are focused on terrestrial ecosystem models, the principles highlighted are general across different systems and processes.

A more predictive global change science needs to be based on ecosystem models that capture important processes rather than merely reproducing patterns (Bonan & Doney, 2018; Lovenduski & Bonan, 2017; Medlyn et al., 2015). Modeling efforts should be geared toward generating hypotheses that are testable against data (Hanson & Walker, 2020). Most current modeling activities, however, are more likely to be informed by high‐volume high‐level observational data (e.g., landscape level biogeochemical fluxes) than experimental manipulations (Wieder et al., 2019) or studies focused on low‐level process details (e.g., interactions between non‐structural carbohydrate reserves, drought, and mortality; Keenan et al., 2013). This is in direct contrast with the incredibly diverse range of data generated by ecology as a discipline (Hanson & Walker, 2020). Until modeling tools become more accessible, new communities of model users who can expand model‐based interpretation and hypothesis testing beyond its limited scope will be curbed by informatics bottlenecks that impede wider representation.

More importantly, current approaches in confronting models with data frequently fail to actively engage the non‐modeler community, who often possess a more detailed understanding of processes and study systems (Jeltsch et al., 2013; Seidl, 2017). This bottleneck not only impacts the pace and the quantity but also the quality of modeling efforts. The division between empirical and modeling research is further exacerbated by the current “uniqueness of models”; that is, each model comes with an idiosyncratic learning curve due to the lack of standards around model interfaces and operation. To restore the balance, we need to concurrently increase modeling literacy and lower the technical barrier for modeling activities (Seidl, 2017). This barrier, overall, hinders efforts to replicate findings, extend analyses to other models and locations, and routinely confront model‐based hypotheses with data (Gil et al., 2016).

We argue that a major step toward reducing these model‐data bottlenecks lies in the development and support of community‐wide cyberinfrastructure: a computational environment where we can effortlessly operate on data, simulate natural phenomena, perform model evaluation, and interpret results (Dietze et al., 2013; Eyring et al., 2019; Gil et al., 2016; also see Appendix A for a glossary of terms). While the general idea is not new, their application has been limited in ecology. However, there are several converging initiatives that make it timely to reinvigorate efforts (see Appendices C and D for example initiatives and their overview, and Box 1).

BOX 1 **How to support and sustain community cyberinfrastructure?**

The ongoing maintenance and development of common cyberinfrastructure tools are essentially conditioned upon uptake and support by the community. This effort typically starts with building a bottom‐up community (Boettiger et al., 2015) involving:

- Support widely adopted languages by the domain scientists (e.g., R and Python) so that:
  - experienced users can get off to a running start,
  - inexperienced users would be motivated to invest efforts with the co‐benefit of learning a popular language,
  - larger communities of these languages can bring further support.
- Initiate strong ties with the demographic that can highly benefit from community solutions such as early career researchers.
- Establish codes of conduct for inclusion and diversity, and encourage participation regardless of experience level.
- Always adhere to open software best practices to build a reputation that can in return attract human resources and funding.

Luckily, these efforts do not need to start from scratch: the community can adopt and build upon existing systems (Appendix C). While we acknowledge that getting involved with community development requires upfront investment of time and resources of individuals, the benefits from participation are significant overall:

- Contributions to community tools perpetuate and increase their value, elevate recognition of their contributors (Dai et al., 2018; Lowndes et al., 2017).
- Community involvement provides larger support and career networks (McKiernan et al., 2016).
- In a research landscape that is ever diversifying, community cyberinfrastructure will be an active learning platform where ecologists gain advanced capability (Dietze et al., 2013).

As the community grows, successful strategies could be taken as an example, such as the WRF (The Weather Research and Forecasting Model) community (Powers et al., 2017):

- Financial and personnel burdens are spread out among the community, while the main support and steering responsibility could remain centralized.
- A help service that is responsible for user assistance is fundamental.
- Building committees in charge of coordination and direction is effective, e.g.:
  - Developers committee, to maintain code design, testing and upkeep.
  - Release committee, to oversee and time major releases.
  - Review committee, for scientific evaluation of major module/package contributions.

Open software and data management plans are increasingly becoming an important requirement by funding agencies (Powers & Hampton, 2019) for which use of community cyberinfrastructure could be fittingly proposed. Thus, we suggest such proposals to include a budget item or person hours for the support of community tools when possible. While projects without funding should also be welcome, short‐term funding opportunities for open research (McKiernan et al., 2016; Powers & Hampton, 2019) will help bottom‐up community building. However, viability over the long‐term requires sustainable funding structures and top‐down support from funding agencies, networks, and the private sector. There are currently several appropriate venues for cyberinfrastructure projects (e.g., NSF Cyberinfrastructure for Sustained Scientific Innovation), but as communities make their cyberinfrastructure needs better known (e.g., through communication with funding agencies and uptake), we expect such opportunities to increase in number and variety. Ultimately, **[R30]** it is important that community and funding agencies support the sustainability of these tools as critical components of the collective scientific infrastructure in a similar way they do with the physical infrastructure (field stations, sensor networks, satellites) and data repositories.

**FIGURE 1:**
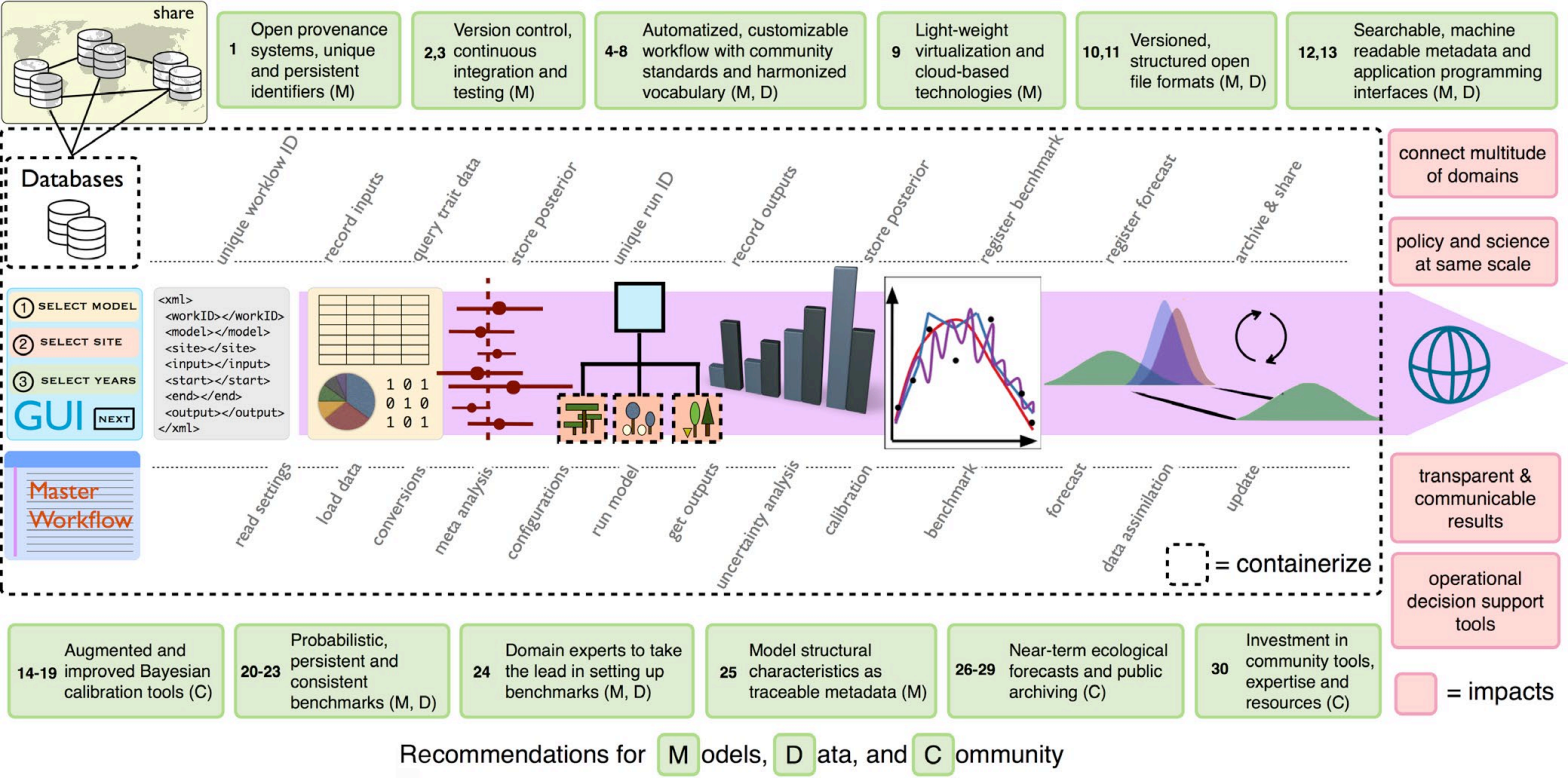
Schematic of a community cyberinfrastructure example and summary of recommendations (numbers in the green boxes refer to our recommendations in the main text). Users start with a high‐level Graphical User Interface (GUI) to provide their setup for a modeling activity. These selections are translated into a human and machine‐readable markup language and read in by the master workflow which then executes a sequence of modularized tasks. At this stage, a unique identifier is assigned to the workflow to be executed. This ID, which points to the full workflow output and access to the metadata required to repeat it, can be shared among collaborators and published in papers. Next, the selections of the user are queried with the database, and actions are decided depending on whether requested items are already processed in an earlier modeling activity and ready to use or need to be retrieved and processed. Then, each module performs a well‐defined task in the specified order. Crucial information for provenance of the whole workflow is recorded in the database during associated steps. Key outputs from analyses, such as calibration posteriors, are stored in a way that enables their exchange and re‐use between different workflows. An important feature of this cyberinfrastructure is that both its parts and itself as a whole are virtualized (containerized) to add an additional layer of abstraction and automation, and to ensure interoperability

In the following sections, we present a roadmap to the key features of a community cyberinfrastructure, and discuss specific challenges and solutions for model‐data activities. These activities include but are not limited to (a) obtaining and processing data (data ingest); (b) estimating model parameters through statistical comparisons between models and real‐world observations (calibration); (c) evaluating and comparing performance skills through standardized and repeatable multi‐model tests (evaluation and benchmarking); and (d) combining model predictions with multiple observations to update our understanding of the state of the system (data assimilation). We provide specific recommendations for the measurement community, the modeler and developer community, and the broader community throughout each section (Figure 1; Appendix B).

## 2 FAIR CYBERINFRASTRUCTURE ESSENTIALS

There should be few things more repeatable in science than running a deterministic model. In practice, running a process‐based simulation model is often fraught with roadblocks to any new user or developer (Dietze et al., 2013). Tackling this at the individual model level leads to redundant efforts across models and inhibits economies of scale that could be gained by sharing informatics tools across communities (for examples of shared ecological informatics infrastructure please see Appendix C). Besides, the larger community of users associated with common infrastructure will foster innovation and create an incentive for developers to make better, more sophisticated algorithms that have gone through more extensive testing (Gil et al., 2016). The revolutionary success of the open source and free programming language R (R Core Team, 2020) aptly exemplifies the importance of community involvement in developing and sharing standard tools for a massive reduction in redundant efforts, as well as having access to a much larger community support (Boettiger et al., 2015; Lai et al., 2019).

**FIGURE 2:**
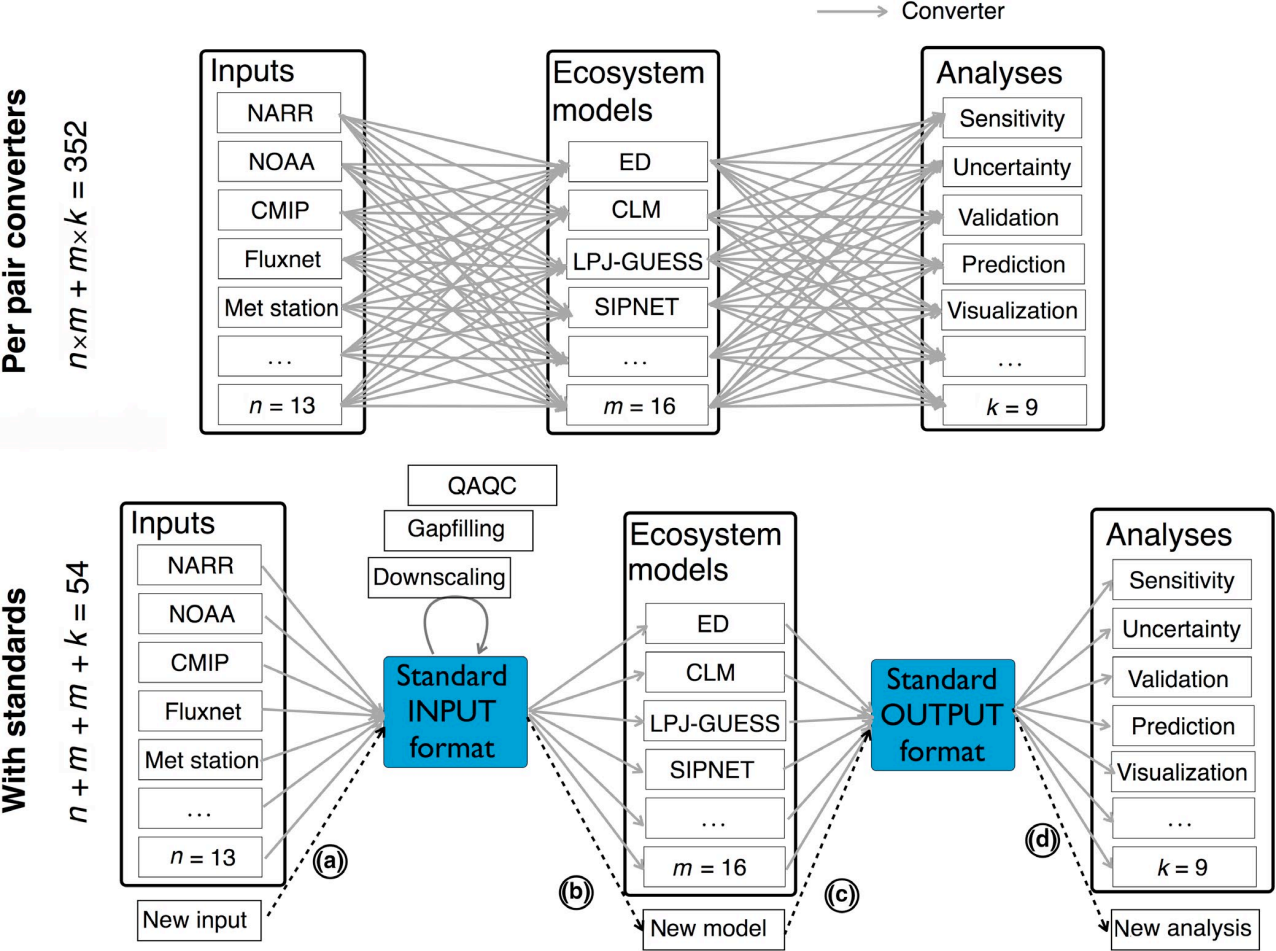
Reduction in redundant work when adopting common formats. There are “*n*” data types that must be linked to “*m*” simulation models and “*k*” post‐simulation analyses. In the top panel, the conventional approach where modeling teams work independently requires implementing *n × m* different input and *m × k* different output conversions. As data, models, and analyses are added, and effort scales quadratically. On the other hand, the bottom panel shows that by working as a community, and adopting common formats and shared analytical tools, the number of converters necessary to link models, data, and analyses reduces to an *m + n* and *m + k* problem, and scales linearly. When a new input source or a new analysis is added to the system, it can immediately get access to *m* models by writing only one converter, (a) and (d) respectively. Likewise, when a new model is added, it can get access to *n* inputs and *k* analyses by writing one converter for each, (b) and (c) respectively. This scaling also extends beyond data conversions to the development of tools and analyses. For example, if input data need to be extracted, downscaled, debiased, gap‐filled, or have their uncertainties estimated, each of these steps does not need *m* × *n* variants but rather just one tool that can be applied to the standard

Here we briefly highlight the FAIR (*findable*, *accessible*, *interoperable*, and *reusable*) cyberinfrastructure essentials to facilitate a catalog of model‐data activities (for more details on FAIR principles for research software and data, please see Culina et al., 2018; Gil et al., 2016; Hasselbring et al., 2020 and the references therein):

- *Findability* refers to the ease with which permanent records of the key metadata about each model‐data activity and computational output can be found (Hasselbring et al., 2020). Recording the full, transparent history of an analysis to enable findability is known as provenance. For large model‐data workflows executing multiple models or experiments, we recommend **[R1; R for recommendation]** model developers utilize open community provenance databases, which assign unique and persistent identifiers to each model‐data activity (Gil et al., 2016; LeBauer et al., 2013). Such identifiers could be used in publications, pointing readers to the full computational output and the metadata required to repeat a model run (Fer et al., 2018). **[R2]** The workflow and provenance system themselves should also be version controlled (e.g., using GitHub) to ensure a fully reproducible record (Piccolo & Frampton, 2016). **[R3]** Then, any changes to their code need to be automatically tested to ensure expected behavior by tools for continuous integration (e.g., Travis CI, travis‐ci.com; Github Actions, github.com/features/actions).
- *Accessibility* in modeling goes beyond obtaining the model code. A broader technical barrier exists in terms of the abilities required to effectively deploy simulation models and perform complex analyses. **[R4]** A well‐defined automated workflow that coordinates individual tasks (Figure 1) should be set up by the developers to (a) reduce barriers to entry; (b) ensure replication is possible; and (c) reduce costs of manual operation. The process of focusing on the design of this workflow, which is also known as abstraction, requires standardizing and generalizing the important tasks involved, and devising how they are related to one another. Leveraging systemized approaches (e.g., tidyverse in R, or pandas in Python) throughout the workflow design promotes consistency, creates predictable expectations, and fosters knowledge transfer across projects. Abstraction further facilitates presenting the user with a **[R5]** more intuitive and accessible interface that handles everything from running ecosystem models in place to submitting complex analyses to remote high‐performance computing resources under the hood.
- *Interoperability* is critical to building cyberinfrastructure that works seamlessly across many models, but this requires predictable file formats for model inputs, outputs, and data constraints used by the community. While reducing the proliferation of both data and model formats would alleviate this in the long term, in the short term **[R6]** using standard data pipelines can remedy the redundant efforts put into building custom tools. For example, consider the common problem of managing the data streams in and out of the models with two cases where (a) every developer team works independently (Figure 2, top panel); and (b) a common pipeline with internal standards is used (Figure 2, bottom panel). Not only is the latter approach much more scalable, but these tools can be made more reliable and sophisticated as less code will be written and tested by more people. **[R7]** We recommend the ecological community leverage existing standard formats as the internal standards, such as the Climate and Forecast convention (Eaton et al., 2017), and the use of ontologies to provide harmonized vocabularies and semantic frameworks (e.g., Stucky et al., 2018).
- *Reusability* of community models and tools builds on interoperability but also requires **[R8]** individual tasks involved be isolated and modularized in the workflow (Figure 1). Modularity would allow (a) internal modifications to their implementation without altering the overall behavior of the system; (b) independent reuse of tools outside of specific systems; and (c) users to swap in/out alternative algorithms/tools and customize their workflow. Community cyberinfrastructure should further be available to users without having to deal with obscure system requirements and dependencies. Similar to what programming language R has achieved, more standardized installation procedures and fewer configuration steps significantly reduce user time for setup and increase adoption, reusability, and reproducibility. Fortunately, modern virtualization technologies offer a number of tools that allow users to run packaged software, called containers, complete with all its dependencies (Piccolo & Frampton, 2016). **[R9]** We recommend developer communities adopt recent light‐weight containerization systems (such as e.g., Docker—www.docker.com; Singularity—singularity.lbl.gov) that are easy to install, set up, upgrade, and scale up with new locations to run the models. Containerization allows existing infrastructures to be run reliably across a variety of computing resources, including cloud‐based virtual services (Farley et al., 2018; Hasselbring et al., 2020).

## 3 DATA INGEST OPPORTUNITIES

Data play a critical role in modeling activities; however, due to their sheer volume and diversity, they can be difficult to locate and obtain as sifting through deluge of data manually is impractical (Reichstein et al., 2019; Waide et al., 2017). **[R10]** To make data FAIR, we recommend data producers use consistent naming structures (e.g., Assistance for Land‐surface Modelling activities convention, also please see Appendix A for more details) and open file formats (e.g., comma‐separated values, netCDF; Hart et al., 2016). **[R11]** Next, data should be stored in data repositories where datasets are versioned, data citations are provided, and that support **[R12]** standard, searchable metadata, and machine‐readable Application Programming Interfaces (e.g., the Oak Ridge National Laboratory Distributed Active Archive Center, Cook et al., 2016; Environmental Data Initiative, Gries et al., 2019; Open Science Framework, Sullivan et al., 2019). When those repositories are part of jointly searchable networks (e.g., DataONE—www.dataone.org), it could further allow developers to leverage one set of tools for many sources.

Admittedly, data providers may have to invest significant time and resources to follow these recommendations. These costs include the following: preparing descriptive metadata to prevent misuse, choosing the right repository with appropriate licensing and without isolating data from relevant disciplines, and finding means (funding and expertise) to manage data especially for small projects (Culina et al., 2018; Gil et al., 2016; Waide et al., 2017). Furthermore, other valid concerns such as data leakage and insufficient recognition are frequently raised (Bond‐Lamberty et al., 2016). While these issues are not specific to the roadmap discussion here, community cyberinfrastructure tools can alleviate them to a certain extent. For example, investments in optimizing standardized protocols, terminologies and file formats for community tools during data collection and processing will help with metadata preparation and repository selection. By getting involved with community cyberinfrastructure, small projects can gain access to larger community expertise and support. Cyberinfrastructure data ingest pipelines can automatically query licenses as chosen by the data provider (Culina et al., 2018) and streamline citations to credit researchers seamlessly. Community tools (such as Brown Dog, browndog.ncsa.illinois.edu) can access and index data collections, in particular small uncurated and/or unstructured data collections, thereby preventing data loss, increasing discovery, and further securing recognition.

On the big data side, approaches for scientifically and computationally interacting with high‐volume, high‐velocity data become increasingly available (Reichstein et al., 2019). While it is important to generalize these cutting‐edge tools and share with the community, modeling activities frequently involve a subset of data (e.g., a specific region or period) for which time to transfer data often exceeds the time to process it. Thus, we endorse the recent paradigm of **[R13]** cloud computing and online services (e.g., Google Earth Engine) that allow users to select, subset, transform, or perform other operations on the data without having to download and expand (see Gomes et al., 2020 for more examples). Within this set up, community cyberinfrastructure also provides a medium where a diverse array of data delivered by Internet of Things techniques can be integrated into models in a sensible manner (Fang et al., 2014). As developers combine cloud‐based cyberinfrastructure tools with cutting‐edge data platforms, this would free the users from their local constraints altogether. Empowering more groups to interact with large datasets brings its own push toward progress in terms of scientific proficiency and diversity (Nagaraj et al., 2020).

## 4 WAY FORWARD IN CALIBRATION

After data ingest, another persistent challenge in process‐based ecosystem modeling is calibration: the process of using data to constrain model parameters (Dietze et al., 2013; Seidel et al., 2018; van Oijen, 2017). Some model parameters may be directly informed by ecological trait data (e.g., turnover rates). In this case, meta‐analysis tools can pull data together from open‐access, machine‐readable, curated databases (LeBauer et al., 2013, 2018; Shiklomanov et al., 2020). A non‐negligible portion of model parameters, however, are often not directly measurable; therefore, there is a need to estimate parameters indirectly using inverse methods that infer what parameter combinations produce model predictions compatible with observations (Hartig et al., 2012). **[R14]** When doing this, we recommend the community take the Bayesian approach to transfer the information from data to probability distributions about models and parameters (Hartig et al., 2012; LeBauer et al., 2013). Bayesian approach allows combining information from multiple sources and scales, iteratively updating our understanding as new data become available, propagating uncertainty into model predictions to inform decision making, and it is becoming more effective in dealing with complex systems with the increase in computing power and numerical methods (van Oijen, 2017).

Most off‐the‐shelf Bayesian tools (e.g., JAGS—mcmc‐jags.sourceforge.net; STAN—mc‐stan.org), however, are not designed to work with external “black box” models. Process‐based models cannot simply be “plugged‐into” these tools and are often too complicated to be re‐implemented in the specific syntax of these software. In addition, **[R15]** these tools need to support re‐reading their own outputs (posteriors) as new inputs (priors), which is critical for iterative updating of the analyses. Due to lack of available tools, models are frequently used uncalibrated (or hand‐tuned; Seidel et al., 2018). Assessment of uncalibrated (or naively calibrated) models can cause poor calibration to be mistaken for inadequate model structure or mask real problems with the model structure, hindering overall progress in model development (van Oijen, 2017). **[R16]** Using multiple data constraints can be critical to ensuring that a model is getting the right answer for the right reason (Medlyn et al., 2015). Even when a model is calibrated for one setting (e.g., site or period), it does not guarantee reliable performance at another setting because there is variability and heterogeneity in natural systems. More flexible techniques, such as hierarchical Bayesian calibration, can formally quantify the scales of unexplained system variability and inform directions for model development (van Oijen, 2017), but there are even fewer available tools for their standard implementation with external models.

Within a community cyberinfrastructure, the challenge of developing advanced calibration tools only needs to be faced by statistics experts. Software alternatives for calibrating “black‐box” models are becoming increasingly available (Fer et al., 2018; Hartig et al., 2019; Huang et al., 2019). **[R17]** Community cyberinfrastructure will be most successful if hierarchical calibration tools are able to account for all kinds of ecological variability and heterogeneity (Farley et al., 2018), and if coupling to a calibration workflow is part of model development. When calibration tools are implemented in community cyberinfrastructure, they can seamlessly link multiple data constraints with multiple models. As such workflows are tracked by provenance systems, **[R18]** results from one analysis (e.g., posteriors) can readily be used by a subsequent analysis elsewhere, accelerating our ability to confront models with data. Investing in such standardization and generalization will not only allow a wider audience to adopt these methods as common practices but also foster progress on **[R19]** developing novel, more advanced calibration techniques (e.g., with emulators, Fer et al., 2018; deep learning, Tao et al., 2020).

## 5 MODEL INTERCOMPARISON AND BENCHMARKING

**FIGURE 3:**
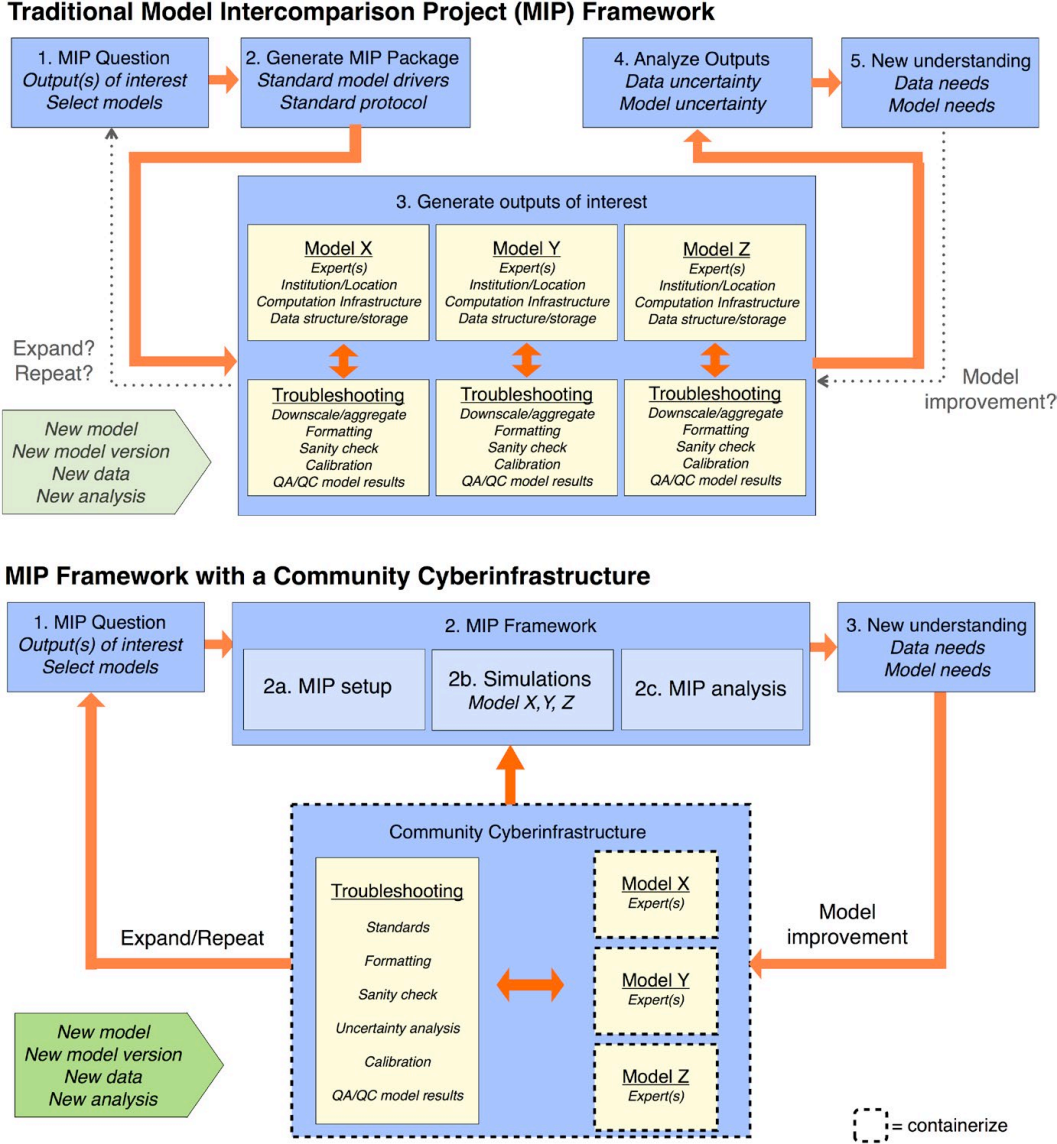
Traditional multi‐model intercomparison project (MIP) workflow versus community cyberinfrastructure. Historically, each model and associated experts/infrastructure individually engage with MIPs (top). While stimulating model improvement is intended, it is not inherently nor readily available in traditional MIPs. In a community cyberinfrastructure, by contrast, both standardization of inputs and outputs and troubleshooting are included in embedding each individual model in the system (bottom) where MIP analyses are a use case. MIP conclusions relevant for model or cyberinfrastructure development can be fed directly back into this framework

Comparing models to data is at the heart of hypothesis testing and model evaluation (Best et al., 2015; Fisher et al., 2014). While process‐based models are frequently compared to multiple datasets across their lifespan, it is remarkably rare to put an ecosystem model through all its past assessment exercises every time it is updated unless a workflow has been automated (Best et al., 2015; Collier et al., 2018). **[R20]** To verify progress, and assess the tradeoffs between model parsimony and complexity, key datasets need to be set as “benchmarks” to track and compare performance through time (Best et al., 2015; Luo et al., 2012). Benchmark data can also be used to compare across models as part of model intercomparison projects (MIPs). However, the lack of automated and shared workflows also makes traditional MIPs logistically challenging to coordinate and repeat (Figure 3, top panel). Modeling groups could face incompatibilities in their results due to differences in their model configurations (e.g., calibrated vs. uncalibrated). Furthermore, due to the cost of performing a MIP, model output requests and experimental designs are typically kept simple. For example, MIPs largely focus on single model realizations which can lead to biased or overprecise decisions about model performances.

Many of the utilities that are particularly valuable for MIPs and benchmarking are already included in embedding each individual model in the community cyberinfrastructure (Figure 3, bottom panel). The use of a cyberinfrastructure also opens up the possibility of more advanced MIP benchmark activities, such as running ensembles to propagate input uncertainty to model output uncertainty. Generating multi‐model ensembles with uncertainties is also practical for studying model structural errors (Bonan & Doney, 2018) and for model averaging which could potentially reduce prediction errors (Dormann et al., 2018). **[R21]** We recommend the community move toward benchmarks that account for model and data uncertainty, and leverage this information when computing model performance scores (e.g., benchmarking that takes into account the uncertainty bounds in models and observations to calculate a score based on overlap probability).

Once a model is integrated into community cyberinfrastructure, it becomes trivial to add its alternative versions, benchmark against existing MIPs and seamlessly feedback to future model developments (Collier et al., 2018; Kelley et al., 2013; Wieder et al., 2019). For example, advancing model versions would benefit from being continually tested against the Free‐Air CO_2_ Experiments (FACE‐MIP; De Kauwe et al., 2014; Hoffman et al., 2017) and the Arctic‐Boreal Vulnerability Experiment (Fisher et al., 2018). Within or in addition to existing frameworks, interactive environments (e.g., Rstudio/Jupyter) would allow users to perform more extensive analyses with pre‐loaded and aligned models and data. However, a number of challenges remain, including how to deal with datasets and metrics that are incomplete or inconsistent with each other (Collier et al., 2018; Hoffman et al., 2017). **[R22]** Thus, we further recommend model developers enable direct comparison to observations when possible. For example, instead of relying on modeled data products (e.g., leaf area index) whose uncertainties are harder to determine, models can be augmented to predict observations (e.g., reflected spectral radiance) as measured by the instruments. In other words, bringing models to data, rather than the other way around, may eventually reduce artificial inconsistencies between datasets that stem from additional manipulations for making data and models match. Concomitantly, community cyberinfrastructure would facilitate **[R23]** interaction with a compilation of standard datasets that models need to be able to reproduce repeatedly (Anderson‐Teixeira et al., 2018; Kraemer et al., 2020; Reyer et al., 2020).

### 5.1 Who sets up benchmarks?

To address the bottleneck that only a small fraction of the data collected by ecologists (often with the aim of improving projections) ever makes its way into ecosystem models and scale up, data generators and disciplinary experts need also be equipped with tools for data‐model comparison, not only the “modeler” minority (Seidl, 2017). Through community cyberinfrastructure, **[R24]** domain experts will more easily be able to compare multiple models to their data and set up persistent benchmarks. For example, with input/output standardization and data harmonization, the person leading the MIP no longer needs to be concerned with multiple file formats and model‐specific terminology while assessing the underlying processes and mechanisms represented in the models. As cyberinfrastructure automates tedious activities associated with a MIP, experts can focus on their analysis rather than the logistics, making modeling activities more relevant for their science.

Yet, even before the challenges of running a model or a MIP, it is nearly impossible for non‐modelers to keep abreast of which models exist, their most updated version, and their respective strengths and weaknesses (Jeltsch et al., 2013; Schwalm et al., 2019). **[R25]** Therefore, we further recommend developers encode model structural characteristics as traceable metadata. Although there are preliminary examples of this (e.g., MsTMIP encoding presence and absence of process representations; Huntzinger et al., 2016), standards need to be developed by the community to provide information about key structural characteristics of models. As a result, process representations that repeatedly perform below average across multiple MIPs can be considered rejected hypotheses (Schwalm et al., 2019), which community cyberinfrastructure could track and in return inform the development of the next generation of models as advancing new hypotheses can regain focus. In time, by centralizing these comparisons into databases, community cyberinfrastructure allows new users to discover new models and to evaluate their updated process representations with minimal technical barriers while allowing the modeling minority to focus on learning from their colleagues and improving models, rather than the status quo where the majority of their time is spent on mundane informatics issues.

## 6 DATA ASSIMILATION AND ECOLOGICAL FORECASTING

For ecology to respond to the pace of global change, and better inform environmental decisions, the nature of the relationship between ecological models and data must be reconsidered. While most ecological analyses tend to be non‐specific and a posteriori (e.g., ANOVA models), and most ecological forecasts are long term (e.g., 2100 projections), there is much to be learned from **[R26]** making near‐term ecological forecasts that can be tested and updated as new observations become available (Dietze et al., 2018; Fox et al., 2009). Adopting an iterative forecasting approach will not only make ecology more relevant to the society, by providing information on fast, decision‐relevant timescales, but will also transform basic ecological science and theory (Dietze et al., 2018), by accelerating the pace at which specific, quantitative, and falsifiable predictions are confronted with data.

Like calibration, the data assimilation methods that drive forecasting, through a formal fusion of data and modeled states (or both states and parameters), also require advanced statistical and computational expertise. Ecological models and data frequently violate the statistical assumptions embedded in assimilation algorithms developed in other disciplines (e.g., normality, homoscedasticity, independence); hence, **[R27]** many existing tools need to be reassessed and generalized by experts within community tools to appropriately meet the ecological model‐data characteristics (Raiho et al., 2020). Making a forecast operational also requires **[R28]** a higher level of repeatability and efficient scheduling of cyclic workflows, where a large number of jobs are executed at regular intervals and each forecast cycle depends on previous ones (Oliver et al., 2019). Overall, the breadth of expertise and investment of resources needed to set up a forecasting pipeline using state‐of‐the‐art data assimilation methods often exceeds the limits of individualistic efforts (White et al., 2019).

Community‐level development of automated pipelines provides a key economy of scale in data assimilation and forecasting and builds upon many of the features already discussed (Dietze et al., 2018): informatics tasks of gathering, processing, and standardizing new data will maximize data use and diversity of contributions. Managing the execution of analytical workflows will refine analyses and make them applicable to new problems. **[R29]** By publicly archiving and reporting results community cyberinfrastructure enables comparisons of different forecasting approaches, future syntheses, and assessment of improvement over time. These features are integral to the vision for such an infrastructure and could then be coupled to, and build upon, existing community tools for workflow scheduling (Oliver et al., 2019) and data assimilation (Fox et al., 2018; Raiho et al., 2020; Pinnington et al., 2020).

## 7 CONCLUSIONS

Scientists, managers, and policymakers increasingly rely on models to understand the impact of decisions on ecological processes (Arneth et al., 2014; Bonan & Doney, 2018; Smith et al., 2019). As the barriers to entry for using the latest models and data are lowered, decisions will be made with better information, and scientific problems will be solved more quickly. Community cyberinfrastructure is the engine to bring time frames associated with model‐data integration in line with the pressing needs of managers, policymakers, and society more broadly. We summarize our major recommendations for promptly meeting the dispersed and variable model‐data synthesis needs of the ecological community as follows.

### 7.1 Integrated community principles and practices

Modeling needs to be open, verifiable, and credible. Three key concepts in modeling cyberinfrastructure—abstraction, automation, and provenance—open up the possibility for realistic replication, community‐wide transparency, and model‐based ecological analysis. Adopting common cyberinfrastructure tools that are accessible, reproducible, interoperable, scalable, and community driven will play a critical role in reshaping how ecologists interact with models.

### 7.2 Reusable data and software

Data processing remains a bottleneck to model improvement. To foster effective discovery and reuse of both data and software, we recommend human‐ and machine‐friendly community‐scale approaches. Developing reusable tools based on community standards and involving the measurement community more deeply in data‐model integration are both essential for scaling up modeling efforts.

### 7.3 More advanced calibration techniques

Testing hypotheses should be done with properly calibrated models. Inconsistencies in model comparison due to different calibration procedures will be reduced by employing shared Bayesian calibration tools that are set up to work with process‐based models. Hierarchical Bayesian calibration solutions and novel algorithms, developed and generalized under community cyberinfrastructure, will help us better capture the inherent variability and heterogeneity in ecological systems.

### 7.4 Persistent benchmarks

Model benchmarking and intercomparison are dynamic activities that need to continually inform model improvement. We recommend a more streamlined, easily repeated, and modified process for benchmarking a suite of models with varying levels of process complexity and scale. Community cyberinfrastructure will allow domain experts to determine and more directly influence the most salient datasets that models need to replicate to demonstrate that they are capturing processes correctly, and then take the lead in setting up and performing these benchmarks.

### 7.5 Near‐term ecological forecasts

Automated data assimilation and forecasting pipelines are a necessity for ecology to support decision‐making in an increasingly non‐equilibrium world that has moved outside of historical norms. Building these forecasting systems requires complex automated systems, and community cyberinfrastructure is well‐positioned for putting the parts of operational forecasts together.

Process‐based models, though imperfect, are our window into the future functioning of ecosystems under global change. The next generation of ecological models will need to ingest increasingly diverse and expansive data to inform and test new process representations and scaling approaches, allow rapid detection and explanation of global change patterns, and even possibly allow them to be prevented. This need is now more pressing than ever. To achieve ecological model‐data integration in a way that is transparent, easily communicable, and scales up to the size and diversity of the ecological community, we must invest in community cyberinfrastructure.

## CONFLICT OF INTEREST

The authors declare no competing interests.

## AUTHOR CONTRIBUTION

All authors were present in the workshop where these ideas were discussed. I.F. and A.K.G. lead the writing with extensive feedback from M.C.D. and with contributions from all authors. All authors have read and approved the manuscript.

## CODE AVAILABILITY

Code availability not applicable to this article. However, we note for the interested reader that all example community tools mentioned in Appendix C are open source and available on online code repositories.

## Supporting information

Supplementary Material

## Data Availability

Data sharing not applicable to this article as no datasets were generated or analyzed during the particular study.
