# Supplementary material for: Beyond ecosystem modeling: A roadmap to community cyberinfrastructure for ecological data‐model integration
Source: Glob Chang Biol. 2020 Nov 6;27(1):13–26. doi: 10.1111/gcb.15409 (PMC7756391; doi:10.1111/gcb.15409)
Supplement: Supplementary file 1 — Supplementary Material [file GCB-27-13-s001.pdf]

Appendix A. Glossary of terms as used in this study

Abstraction is the process of focusing on the design of the information technology system architecture, how components are related to one another and details of their overall functioning.

Accessibility of software and data requires either the complete metadata, or the data and the software themselves to be published in a publicly accessible location. In addition, the protocols to be able to use, install, implement and employ should be shared.

Algorithms are a well-defined set of rules to solve a computational problem.

Application Programming Interfaces (APIs) are tools to programmatically interact with data online.

Assistance for Land-surface Modelling activities (ALMA) is a data convention to provide consistent name, unit and sign standards for variables to facilitate stable exchange among various model-data activities.

Benchmarking is a way of assessing the skill of a model with a priori performance expectations.

Comma-separated values (CSV) are plain delimited text files that use commas to separate values.

Cyberinfrastructure is an information technology system that consists of multiple components from data storage to advanced informatics tools to operate on data, simulate natural phenomena, perform model evaluation and scientific interpretation.

Data assimilation is an approach for combining model predictions with multiple observations to update our understanding of the state of the system.

Database is a structured electronic system that typically stores and organizes data and metadata.

FAIR: findable, accessible, interoperable and reusable

Findability is the amenity of data and software with respect to their discoverability by both humans and machines. This requires assigning globally unique and persistent identifiers pointing to a rich metadata about data, software and all the computational activity around them.

Internet of Things (IoT) in ecology and environmental science context refers to a network of interconnected sensors and smart devices for automated field-based environmental monitoring, data collection and coordination without human interaction.

Interoperability is an attribute of a software or collection of software that refers to its compatibility with other products.

Metadata is a set of descriptive and informative data that provides documentation about the data or software or the computational activity around them.

Model Intercomparison Projects (MIPs) are collective, typically multi-institutional model evaluation and benchmarking efforts to enhance scientific understanding and stimulate model improvement.

Network Common Data From (NetCDF) is a data file format that is capable of storing and organizing array-oriented scientific data types together with their metadata.

Ontologies are standardized, harmonized, controlled formal collection of terms.

Open file format is a file format that could be read, written, used and implemented by anyone and any file processing software without any restrictions. Good examples are CSV and netCDF files that can be imported and exported by many software.

Provenance is the complete historical information of an analysis about all steps and components involved in producing it.

Repository is an electronic, typically online, storage for hosting software and scientific code, ideally with related documentation and meta-data.

Reusability is the versatility of existing data and software to be reused, replicated, combined, adapted and extended in different settings.

Scheduler is a software for automatic managing scientific workflows periodically.

Software container is a package of an application with all its dependencies that enables the execution of the code in many computing environments.

Workflow is a series of computational tasks that are executed in an order to assemble multiple activities with data flow in-between.

Virtual machines are software that provide all the basic functionality of an operating system in a contained environment.

Appendix B. Table of recommendations for the modeler and developer community (M), the measurement and data community (D) and the broader community (C).

#	Recommendations	For
**R1**	Systematic open community provenance systems, unique and persistent identifiers	M
**R2**	Version control for both the software and the provenance system	M
**R3**	Continuous integration and testing	M
**R4**	Automated, standardized and generalized workflow	M
**R5**	Intuitive and accessible graphical user interfaces	M
**R6**	Well-defined internal standard formats	M
**R7**	Use of harmonized vocabulary, units and semantic frameworks	M, D
**R8**	Isolated and modularized tasks for customizable workflow	M
**R9**	Virtualization using light-weight containerization systems	M
**R10**	Consistent structure and open file formats	M, D
**R11**	Curated, versioned data sets with citations provided	D
**R12**	Machine-readable metadata for data sets, Application Programming Interfaces for tools	M, D
**R13**	Expanded services to transform and operate on data online and on the cloud	M
**R14**	Bayesian approach to treat and report uncertainties as probability distributions	C
**R15**	Tools that can re-read their own outputs (posteriors) as new inputs (priors)	M
**R16**	Use of multiple data constraints in calibration	M
**R17**	Hierarchical Bayesian calibration tools to account for ecological variability and heterogeneity	M
**R18**	Automated sharing of posteriors and analysis results for subsequent analyses	M
**R19**	Faster and novel calibration algorithms	M
**R20**	Model performance tracked and compared through time	M
**R21**	Multi-model ensembles with uncertainties and probabilistic benchmark scores	M
**R22**	Model outputs that are directly comparable to observations as measured by the instruments	M
**R23**	Standard datasets as persistent benchmarks	M, D
**R24**	Domain experts to take the lead in benchmarking and model intercomparison projects	C
**R25**	Model structural characteristics encoded as traceable metadata	M
**R26**	Near-term ecological forecasts	C
**R27**	Generalized data-assimilation techniques compatible with ecological assumptions	M
**R28**	Efficient scheduling of cyclic workflows	M
**R29**	Public archiving and reporting of forecasts	M
**R30**	Best practices for sustainable community tools, expertise and resources (see Box in main text)	C

Appendix C. Examples of open source projects and initiatives with multi-model analysis capabilities

- **Data Assimilation Research Testbed (DART)** is a community facility for data assimilation (Anderson et al., 2009). Website: www.image.ucar.edu/DAReS/DART/, GitHub: https://github.com/NCAR/DART, Last access: 2020-10-13 Also see CIME (Common Infrastructure for Modeling the Earth) and DART coupling GitHub: https://github.com/ESMCI/cime
- **Ecological Platform for Assimilating Data (EcoPAD)** is an interactive system for model-data fusion (Huang et al. 2019) Website: https://ecolab.nau.edu/ecopad/, GitHub: https://github.com/ou-ecolab, Last access: 2020-10-13
- **Earth System Model Evaluation Tool (ESMValTool)** is a community tool for evaluation of Earth System Models (Righi et al., 2020, Eyring et al. 2020). Website: https://www.esmvaltool.org, GitHub: https://github.com/ESMValGroup/ESMValTool, Last access: 2020-10-13
- **International Land Model Benchmarking (ILAMB)** is a model-data intercomparison and integration project (Collier et al., 2018) Website: https://www.ilamb.org/, GitHub: https://github.com/rubisco-sfa/ILAMB, Last access: 2020-10-13
- The **Land Variational Ensemble Data Assimilation Framework** (LAVENDAR, Pinnington et al., 2020) is a tool that implements the method of Four-Dimensional Ensemble Variational (4DEnVar) data assimilation for land surface models. It is also built using Rose and Cylc to control all the model runs (for more information also see: https://jules-lsm.github.io/tutorial/bg_info/tutorial_julesrose/jr_user.html) GitHub: https://github.com/pyearthsci/lavendar, Last access: 2020-10-13
- NASA software suite: **Land Surface Data Toolkit** (LDT, Arsenault et al. 2018) is a model and data pre-processing environment for **Land Information System** (LIS, GitHub: https://github.com/NASA-LIS/LISF) which encapsulates model-data fusion tools. LDT and LIS are part of a software suite together with **Land Surface Verification Toolkit** (LVT, Kumar et al., 2012) that further performs verification and benchmarking of the models https://lis.gsfc.nasa.gov/, Last access: 2020-10-13
- **Parameter ESTimation (PEST)** and Uncertainty analysis is a model independent software for calibration and data assimilation. Website: pesthomepage.org, GitHub: https://github.com/usgs/pestpp, Last access: 2020-10-13
- **Predictive Ecosystem Analyzer (PEcAn)** is an ecological informatics toolbox for model-data fusion and forecasting. Website: pecanproject.org, GitHub: https://github.com/PecanProject, Last access: 2020-10-13
- **Protocol for the Analysis of Land Surface Models (PALS)** is a web application for evaluating land surface/ecosystem models (Abramowitz, 2012) Website: modelevaluation.org, GitLab: https://gitlab.com/modelevaluation/me.org-r-library, Last access: 2020-10-13

In addition, please also see review by Xia et al. (2019) for various land data assimilation systems.

Table A1: Examples of open source projects and initiatives with multi-model analysis capabilities. “Not applicable” fields exempt tools that are meant to fulfill only specific tasks (e.g. ILAMB for benchmarking) or tools that have not incorporated certain tasks yet. Broader patterns emerging from the figure suggest that the community is strong in following certain recommendations, such as version controlling and using open file formats, whereas there is still much to be done for achieving some goals such as developing hierarchical Bayesian approaches and model metadata encoding standards.

Legend: Yes; No/unknown; Not applicable

	FAIRness									Data Ingest			Calibration						Benchmarking				Data assimilation			
	R1: Open provenance system	R2: Version control	R3: Continuous integration	R4: Standardized and automated workflow	R5: Graphical User Interfaces	R6: Intermediate standard	R7: Harmonized vocabulary	R8: Modularized tasks	R9: Virtualization, containerization	R10: Structured and open file formats	R12: API for the software	R13: Cloud operations	R14: Bayesian calibration	R15: Iterative tools	R16: Multiple data constraints	R17: Hierarchical Bayes	R18: Automated posterior sharing	R19: Novel approaches	R20: Track model performance	R21: Multi-model uncertainties	R23: Benchmark datasets	R25: Model metadata encoding	R26: Near term forecasts	R27: SDA algorithms	R28: Cycling scheduling	R29: Public archiving
Data Assimilation Research Testbed (DART)	No/unknown	Yes	No/unknown	Yes	No/unknown	Yes	Yes	Yes	No/unknown	Yes	No/unknown	No/unknown	Not applicable	Not applicable	Not applicable	Not applicable	Not applicable	Not applicable	Not applicable	Not applicable	Not applicable	Not applicable	Yes	Yes	Yes	No/unknown
Ecological Platform for Assimilating Data (EcoPAD)	Yes	Yes	Yes	Yes	Yes	No/unknown	Yes	Yes	Yes	Yes	Yes	Yes	Yes	Yes	Yes	No/unknown	Yes	Yes	Yes	Yes	Yes	No/unknown	Yes	No/unknown	Yes	Yes
Earth System Model Evaluation Tool (ESMValTool)	Yes	Yes	Yes	Yes	No/unknown	Yes	Yes	Yes	Yes	Yes	Yes	Yes	Not applicable	Not applicable	Not applicable	Not applicable	Not applicable	Not applicable	Yes	Yes	Yes	No/unknown	Not applicable	Not applicable	Not applicable	Not applicable
International Land Model Benchmarking (ILAMB)	No/unknown	Yes	Yes	No/unknown	No/unknown	No/unknown	Yes	No/unknown	No/unknown	Yes	No/unknown	No/unknown	Not applicable	Not applicable	Not applicable	Not applicable	Not applicable	Not applicable	Yes	Yes	Yes	No/unknown	Not applicable	Not applicable	Not applicable	Not applicable
Land Variational Ensemble Data Assimilation Framework (LAVENDAR)	No/unknown	Yes	No/unknown	Yes	No/unknown	No/unknown	No/unknown	Yes	No/unknown	Yes	No/unknown	Yes	Yes	Yes	Yes	No/unknown	No/unknown	Yes	Not applicable	Not applicable	Not applicable	Not applicable	Yes	Yes	Yes	Yes
NASA software suite (LDT + LIS + LVT)	No/unknown	Yes	No/unknown	Yes	No/unknown	Yes	Yes	Yes	No/unknown	Yes	No/unknown	Yes	Yes	Yes	Yes	No/unknown	No/unknown	Yes	Yes	Yes	Yes	No/unknown	Yes	Yes	Yes	No/unknown
Parameter Estimation & Uncertainty Analysis (PEST)	No/unknown	Yes	Yes	Yes	Yes	Yes	Yes	Yes	Yes	Yes	Yes	Yes	Yes	Yes	Yes	No/unknown	Yes	Yes	Not applicable	Not applicable	Not applicable	Not applicable	Yes	Yes	Yes	Yes
Predictive Ecosystem Analyzer (PEcAn)	Yes	Yes	Yes	Yes	Yes	Yes	Yes	Yes	Yes	Yes	Yes	Yes	Yes	Yes	Yes	Yes	Yes	Yes	Yes	Yes	Yes	No/unknown	Yes	Yes	Yes	Yes
Protocol for the Analysis of Land Surface Models (PALS)	Yes	Yes	Yes	Not applicable	Yes	Yes	Yes	Not applicable	Yes	Yes	Yes	No/unknown	Not applicable	Not applicable	Not applicable	Not applicable	Not applicable	Not applicable	Yes	Yes	Yes	No/unknown	Not applicable	Not applicable	Not applicable	Not applicable

API: Application Programming Interface here refers to a computing interface for the software itself to interact with its functionalities (as opposed to its capability to interact with other APIs).
SDA: State Data Assimilation here refers to not just batch methods but also Kalman filter and variational methods.
Table includes the recommendations that are for only the modeler and developer community as given on Appendix B.
We also note that some of these software are not necessarily designed as stand-alone tools but could be coupled with other operational systems (e.g. DART + CIME).

Appendix D. The PEcAn Project example

The authors of this paper came together in a workshop that was organized by the PEcAn Project (Predictive Ecosystem Analyzer, pecanproject.org) (Fig A1). Therefore, we expand on the PEcAn example here some more for the interested reader. The PEcAn Project started with National Science Foundation support in 2011. Since then, >50 developers have contributed code and 32 versions of the PEcAn software have been released. New code additions are reviewed by peers and are not incorporated unless they are documented thoroughly for both developers and users. Every submission goes through a set of automatic tests to verify that the code is working as expected. Significant revisions, additions and releases are discussed and agreed upon in online and offline meetings. Stable code release is available through Github, as a fully integrated Virtual Machine (VM), and as a collection of containers on DockerHub. These platforms provide a variety of options to access the PEcAn software, use it to its fullest, and facilitate user feedback.

To help introduce new developers to the community, PEcAn has been participating in the Google Summer of Code (summerofcode.withgoogle.com) since 2017. PEcAn has also been used in many short courses (e.g. 'Summer Course in Flux Measurements and Advanced Modeling' (http://www.fluxcourse.org) and the PalEON summer course 'Assimilating long-term data into ecosystem models', http://www.paleonproject.org) and workshops where new users gain first hand experience. Over the years, the PEcAn Project was supported by numerous grants to the community (both to the users and the developers) from funding agencies and industry partners around the world (e.g. NSF Advances in Bioinformatics, NASA Terrestrial Ecosystems, Amazon AWS in Education Grant etc.). As a result, PEcAn tools were used in numerous scientific publications, and presentations at national and international seminars and meetings[1].

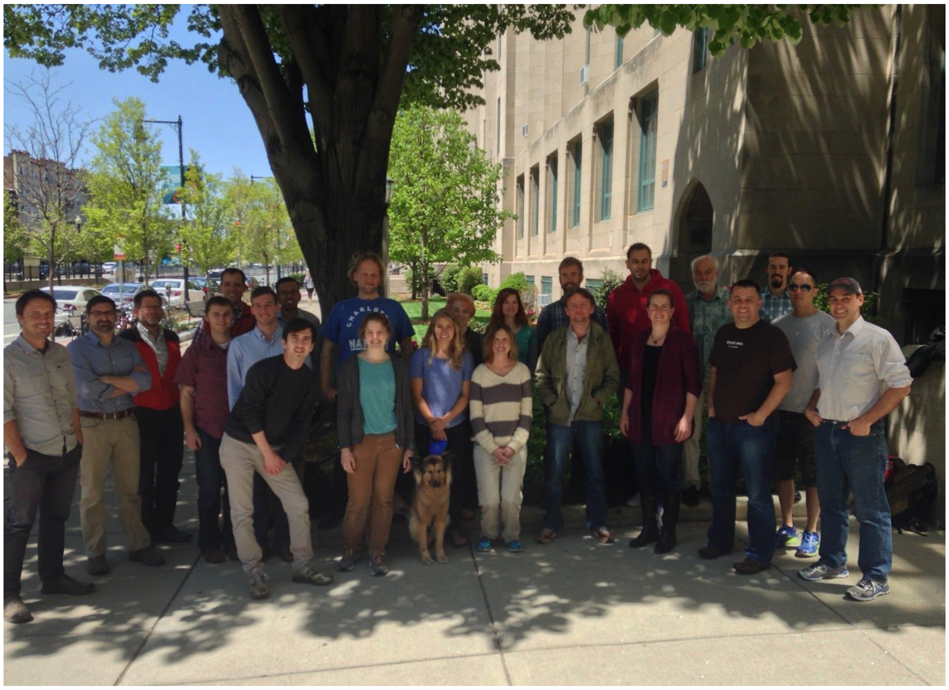

Fig A1. PEcAn workshop group photo, Boston University, USA, 2016.

[1] https://scholar.google.com/citations?hl=en&user=HWhxBY4AAAAJ

Cited references

Abramowitz G. (2012). Towards a public, standardized, diagnostic benchmarking system for land surface models. *Geosci. Model Dev.* **5**: 819–827. doi:10.5194/gmd-5-819-2012.

Anderson J, Hoar T, Raeder K, Liu H, Collins N, Torn R, and Avellano A. (2009). The data assimilation research testbed: A community facility. Bulletin of the American Meteorological Society, 90(9), 1283–1296. https://doi.org/10.1175/2009BAMS2618.1

Arsenault KR, Kumar SV, Geiger JV, et al. (2018). The Land surface Data Toolkit (LDT v7.2) – a data fusion environment for land data assimilation systems. *Geosci. Model Dev.* **11:** 3605–3621, https://doi.org/10.5194/gmd-11-3605-2018

Collier N, Hoffman FM, Lawrence DM, et al. (2018). The International Land Model Benchmarking (ILAMB) system: Design, theory, and implementation. *Journal of Advances in Modeling Earth Systems*. **10:** 2731– 2754. https://doi.org/10.1029/2018MS001354

Eyring V, Bock L, Lauer A, Righi M, Schlund M, Andela B, et al. (2020). Earth System Model Evaluation Tool (ESMValTool) v2.0 – an extended set of large-scale diagnostics for quasi-operational and comprehensive evaluation of Earth system models in CMIP, Geosci. Model Dev., 13, 3383–3438, https://doi.org/10.5194/gmd-13-3383-2020

Huang Y, Stacy M, Jiang J, et al. (2019). Realized ecological forecast through an interactive Ecological Platform for Assimilating Data (EcoPAD, v1.0) into models. *Geosci. Model Dev.* **12:** 1119–1137. https://doi.org/10.5194/gmd-12-1119-2019

Kumar SV, Peters-Lidard CD, Santanello J, Harrison K, Liu Y, and Shaw M. (2012). Land surface Verification Toolkit (LVT) — A generalized framework for land surface model evaluation, Geosci. Model Dev., 5(3), 869–886, doi:10.5194/gmd-5-869-2012.

Pinnington E, Quaife T, Lawless A, Williams K, Arkebauer T, and Scoby D. (2020). The Land Variational Ensemble Data Assimilation Framework: LAVENDAR v1.0.0, Geosci. Model Dev., 13, 55–69, https://doi.org/10.5194/gmd-13-55-2020.

Righi, M., Andela, B., Eyring, V., Lauer, A., Predoi, V., Schlund, M., Vegas-Regidor, J., Bock, L., Brötz, B., de Mora, L., Diblen, F., Dreyer, L., Drost, N., Earnshaw, P., Hassler, B., Koldunov, N., Little, B., Loosveldt Tomas, S., and Zimmermann, K. (2020). Earth System Model Evaluation Tool (ESMValTool) v2.0 – technical overview, Geosci. Model Dev., 13, 1179–1199, https://doi.org/10.5194/gmd-13-1179-2020.

Xia, Y., Hao, Z., Shi, C. *et al.* (2019). Regional and Global Land Data Assimilation Systems: Innovations, Challenges, and Prospects. *J Meteorol Res* **33,** 159–189. https://doi.org/10.1007/s13351-019-8172-4
